# Environmental enrichment accelerates ocular dominance plasticity in mouse visual cortex whereas transfer to standard cages resulted in a rapid loss of increased plasticity

**DOI:** 10.1371/journal.pone.0186999

**Published:** 2017-10-26

**Authors:** Evgenia Kalogeraki, Justyna Pielecka-Fortuna, Siegrid Löwel

**Affiliations:** 1 Department of Systems Neuroscience, J.F.B. Institut für Zoologie und Anthropologie, Universität Göttingen, Göttingen, Germany; 2 Göttingen Graduate School of Neurosciences, Biophysics and Molecular Biosciences (GGNB), Göttingen, Germany; University Medical Center Goettingen, GERMANY

## Abstract

In standard cage (SC) raised mice, experience-dependent ocular dominance (OD) plasticity in the primary visual cortex (V1) rapidly declines with age: in postnatal day 25–35 (critical period) mice, 4 days of monocular deprivation (MD) are sufficient to induce OD-shifts towards the open eye; thereafter, 7 days of MD are needed. Beyond postnatal day 110, even 14 days of MD failed to induce OD-plasticity in mouse V1. In contrast, mice raised in a so-called “enriched environment” (EE), exhibit lifelong OD-plasticity. EE-mice have more voluntary physical exercise (running wheels), and experience more social interactions (bigger housing groups) and more cognitive stimulation (regularly changed labyrinths or toys). Whether experience-dependent shifts of V1-activation happen faster in EE-mice and how long the plasticity promoting effect would persist after transferring EE-mice back to SCs has not yet been investigated. To this end, we used intrinsic signal optical imaging to visualize V1-activation i) before and after MD in EE-mice of different age groups (from 1–9 months), and ii) after transferring mice back to SCs after postnatal day 130. Already after 2 days of MD, and thus much faster than in SC-mice, EE-mice of all tested age groups displayed a significant OD-shift towards the open eye. Transfer of EE-mice to SCs immediately abolished OD-plasticity: already after 1 week of SC-housing and MD, OD-shifts could no longer be visualized. In an attempt to rescue abolished OD-plasticity of these mice, we either administered the anti-depressant fluoxetine (in drinking water) or supplied a running wheel in the SCs. OD-plasticity was only rescued for the running wheel- mice. Altogether our results show that raising mice in less deprived environments like large EE-cages strongly accelerates experience-dependent changes in V1-activation compared to the impoverished SC-raising. Furthermore, preventing voluntary physical exercise of EE-mice in adulthood immediately precludes OD-shifts in V1.

## Introduction

It is well-established that environmental/rearing conditions exert a massive influence on brain plasticity, both in the healthy and stroke-affected brain [1]. Enriched environment (EE) cages provide increased physical (running wheels), social (bigger housing groups), and cognitive (regularly changed labyrinths or toys) stimulation compared to the deprived rearing conditions of standard cages (SC) [2]. The brains of rodents raised in these “enriched” or less deprived cages are markedly different from those of SC-raised animals in numerous ways ranging from molecular to anatomic and functional levels [3]. Housing rats and mice in EE-cages has been shown to enhance plasticity of the primary visual cortex (V1) [4–7], protects against lesion-induced impairments of V1-plasticity and preserves ocular dominance (OD) plasticity not only into adulthood [8] but even lifelong [9].

Occluding one eye of vision (monocular deprivation, MD) in kittens during a brief period in early postnatal life resulted in a drastically changed OD in their V1 [10]. This observation made OD-plasticity one of the best studied models of experience-dependent plasticity in the mammalian cortex [10, 11]. While the binocular visual cortex of mice shows stronger responses to stimulation of the contralateral eye and weaker responses to ipsilateral eye stimulation [12–15], depriving the stronger contralateral eye of vision nevertheless causes a change in the dominance so that V1-neurons get activated more similarly by stimulation of each eye [12, 16].

OD-plasticity in primary visual cortex of SC-raised C57BL/6J mice is age-dependent: it is maximal at 4 weeks (wk) of age, declines after 2–3 months, and is usually absent beyond postnatal day 110 (P110). In 4-wk-old SC-mice (critical period), 4 days (d) of MD are sufficient to induce an OD-shift towards the open eye [16–20]. This juvenile OD-shift is predominantly mediated by a decrease in the visual cortical responses to the deprived eye [11, 18, 21], whereas significant OD-shifts in young adult SC-mice (P60-90) need 7 days of MD and are mediated primarily by a delayed increase of open eye responses in V1 [19, 20, 22]. Beyond that age, even 14 days of MD have not reliably induced OD-plasticity [18, 19].

Restoration of OD-plasticity in older ages is of a particular interest not only for keeping the brain in a more juvenile state and therefore more plastic, facilitating learning and memory, but also has great potential for therapeutic rehabilitation and recovery from injury in the adult brain. Several manipulations to alter the animal’s environment have been tested for the possibility to restore OD-plasticity in the adult brain. One of the most interesting non-pharmacological approaches is the enrichment of the housing environment. Exposing or raising animals in a more stimulating environment compared to the restricted SC-housing promoted OD-plasticity in adulthood [5, 8]. Enriched environment is classically defined as “a combination of complex inanimate and social stimulation” [2]. Compared to the simple SC-cages in which the animals are housed in small groups (up to 5 animals) or even alone with only nesting material, food, and water, EE cages are larger with the mice housed in bigger groups (up to 16 animals) and equipped with a variety of stimulating objects (e.g. running wheels, maze, tunnels, nesting material and stairs). Thus, EE-cages provide animals with strongly increased possibilities for social interactions, physical exercise, exploration and cognitive stimulation [23].

As previously described, raising mice in EE prolonged the sensitive phase of OD-plasticity in mice older than P110 [8]. Here, we investigated whether shorter MD-duration might also be sufficient to induce an OD-shift in EE-mice of 3 different age groups (critical period: P24-35, young: P90-104 and adult: P117-283). Indeed, we observed that already after 2 days of MD, EE-mice of all analyzed age groups showed significant OD-shifts in V1 towards the open eye, which were getting stronger after longer deprivation periods.

While OD-plasticity was restored in mice and rats transferred from SC- to EE cages [5, 8], it had not yet been analyzed whether transferring EE-mice back to more deprived SCs will result in changes of OD-plasticity. To this end, EE-raised mice (>P130) where transferred to SCs and received an MD after 1 d or 1 wk of SC-housing. Notably, OD-plasticity was rapidly abolished, even after only one week of SC-housing. One of the molecules that are changed by EE-raising compared to SC-housing is serotonin [3]: serotonin levels were found elevated in EE-mice and administration of the selective serotonin reuptake inhibitor fluoxetine could mimic the effects of EE [24]. We thus attempted to prevent the loss of OD-plasticity in EE-mice transferred to SC-cages (EE→SC) by supplementing them with fluoxetine (to keep serotonin levels elevated). Finally, another group of EE-mice was transferred to SCs that were equipped with a running wheel (RW) to test whether running might be sufficient to prevent the OD-plasticity loss in the EE→SC-mice. While fluoxetine treatment had no effect on preserving OD-plasticity, the EE→SC-mice with access to a RW continued to display OD-plasticity.

## Material and methods

### Animals and rearing conditions

All mice were from the mouse colony of the central animal facility of the University Medical Center Göttingen (UMG), and housed with a 12h light/dark cycle, with food and water available ad libitum. All experimental procedures were approved by the local government (Niedersächsisches Landesamt für Verbraucherschutz und Lebensmittelsicherheit), and the experimental procedures also comply with National Institutes of Health guidelines for the use of animals.

#### EE-groups

Female mice of three different age groups born and raised in EE were used. Group 1 consisted of 26 critical period mice (P24-35; EE critical period), group 2 consisted of 20 young adult mice (P90-104; EE young) and group 3 consisted of 38 mice older than P110 (P117-283; EE old). About half of the animals underwent monocular deprivation (MD) for either 1, 2, 4 or 7 days followed by daily measurements in the optomotor setup and optical imaging of intrinsic signals. Mice without MD served as controls. Intrinsic imaging data of 7dMD-old mice were reanalyzed from Greifzu, Pielecka-Fortuna [8].

Three additional groups of mice were transferred from EE- to SC-cages after P130 (EE→SC-mice):

#### Group 1

8 female mice between P135 and P278 born and raised in EE were transferred to SCs for 1 day (EE→SC_1d_) or 1 week (EE→SC_1w_) before MD was performed for 7 days. Before and during MD, the spatial frequency and contrast sensitivity thresholds of the optomotor reflex were measured daily by using optomotry, followed by optical imaging on the 7th day.

#### Group 2 (EE→SC fluoxetine/water)

Another group of EE→SC mice were treated with fluoxetine, a selective serotonin reuptake inhibitor (Fluoxetine hydrochloride, Tocris bioscience), administrated through the drinking water [25]. This group consisted of 7 female mice transferred to SCs between P266 and P294; mice were housed in groups of 3 to 5 animals per SC-cage and fluoxetine was given to them immediately after transfer for a period of 3 weeks. After 2 weeks in SC, about half of the mice underwent MD, then spatial vision was measured daily by optomotry as in the EE→SC-mice (above). In order to reach an average daily intake of 10 mg/kg fluoxetine per mouse, a dosage that was described to have an antidepressive effect in mice [25], the concentration of the drug in the drinking water was calculated based on the average daily drinking amount of mice (5ml) [26] and the average mouse weight (25g, JAX^®^ Mice, Clinical & Research Services). Thus, the concentration of fluoxetine in the drinking water was 0.05 mg/ml. The solution was prepared fresh every day and the average consumption was measured. Dripping-free bottles (Bioscape GmbH, Castrop-Rauxel) were used for that propose. For control, a group of EE→SC-mice from the same litter, but drinking only water (EE→SC_-fluox_) were used (n = 7). As mice were housed in groups of 3 to 5 animals per cage the amount of water consumed per cage was divided by the number of the mice housed in every cage to calculate the average water consumption per mouse per day.

#### Group 3 (EE→SC_RW_)

3 adult female mice (P198-211) raised in EE were transferred to a SC (for exact size see below) with a RW for 3 weeks. A 7 day MD was performed after 2 weeks. Afterwards the same procedures as for the group above were followed.

### Housing conditions

#### Enriched environment cages

Our”standardized” EE cages (Marlau, Viewpoint, France; 56×37×32 cm [L×W×H]) are about nine times larger than conventional SCs (26×20×14 cm [L×W×H]), with two floors linked by a ladder for going up and a tube for sliding down. On the lower compartment is the “living area” with three running wheels for physical exercise, a red tunnel to protect the animals from light, and the “food area” where the mice can find food. In order to move from the “living area” to the “food area”, mice have to go to the upper compartment using the ladder, pass through the maze and slide down. They can return to the “living area” through a revolving door which opens only in one direction, thus they are forced to move through the maze again in order to get food. The maze was changed three times per week, and there were in total 12 different configurations. Additionally, mice in EE had more social interactions as they were housed in bigger groups with up to 16 mice per cage compare to 3 to 4 mice per SC. Pregnant females were put into the EE cages one week before delivery (5–7 days). Pups were separated from their mothers and placed in separate female and male groups at P30.

#### Running wheel cages

For experiments with mice in cages equipped with a running wheel (RW) EE-mice were transferred to SCs that were slightly larger than conventional SCs (43×27×19 cm [L×W×H]) to fit a running wheel. The number of RW-turns was counted daily using a counter bind on the wheel and the average RW-turns per animal/per day was calculated.

### Monocular deprivation (MD)

The right eye of the mice was deprived for 1, 2, 4 or 7 days according to published protocols [8, 16]. Briefly, mice were box anesthetized with 2% isoflurane in O_2_/N_2_O (1:1), eyelids were closed with two sutures. Mice were returned to their home cages for recovery and checked daily to ensure that the eye remained closed. In case of an open MD-eye, mice were excluded from further experiments. MD-eyes were opened immediately before the imaging experiments.

### Virtual-reality optomotor setup

Both the spatial frequency threshold and the contrast sensitivity threshold of the optomotor reflex were measured using the virtual-reality optomotor system [27]. Briefly, freely moving mice were positioned on a small platform surrounded by four computer monitors (33.5x26.5cm) forming a box. Mirrors placed at the bottom and the top are giving the impression of an endless cylinder. A rotating virtual cylinder, composed of a vertical sine wave grating, is projected on the screens. Parameters like spatial frequency, contrast and speed of the moving sine wave grating can be varied by the experimenter. In case the mouse can detect the stimulus, it is reflexively tracking the grating by moving the head in the direction of rotation. Spatial frequency thresholds at full contrast and contrast thresholds at six different spatial frequencies [0.031, 0.064, 0.092, 0.103, 0.192, 0.272 cycles/degree (cyc/deg)] were measured daily (after MD/ noMD). For baseline values, spatial frequency and contrast sensitivity thresholds were measured on day 0 (before MD) for both eyes. Since values were not different between the eyes (p>0.05 for all comparisons, ANOVA), left and right eye values were averaged for further analyses.

### Optical imaging of intrinsic signals and visual stimuli

#### Surgery

As described previously in Kalogeraki, Greifzu [28]. Shortly, mice were box-anesthetized with 2% halothane in O_2_/N_2_O (1:1) and injected with atropine (0.1mg/mouse s.c.; Franz Köhler), dexamethasone (0.2mg/mouse s.c.; Ratiopharm), and chlorprothixene (0.2mg/mouse i.m.; Sigma) and rimadyl (0.1mg/mouse s.c.; Zoetis). After placing mice in a stereotaxic frame, anesthesia was maintained with 0.8% halothane in a 1:1 mixture of O_2_/N_2_O through an inhalation mask. An incision of the skin was made over the visual cortex and low-melting point agarose (2.5% in 0.9% NaCl) and a glass coverslip were placed over the exposed area.

#### Data acquisition and visual stimulation

Mouse V1-responses were recorded through the skull using the Fourier imaging technique (29), optimized for the assessment of OD-plasticity (30). V1-signals were visualized with a CCD-camera (Dalsa® 1M30) using a 135x50mm tandem lens configuration (Nikon), with red illumination light (610±10nm). Active brain regions absorb more of the red light and appear darker in the images. Frames were acquired at a rate of 30Hz, temporally binned to 7.5Hz, and stored as 512x512 pixel images after spatial binning of the camera image (pixel size: 8.9μm).

Visual stimuli were presented on a high refresh rate CRT monitor (Hitachi, ACCUVUE, HM-4921-D, 21”, 85Hz) positioned 25cm from the eyes. Stimuli consisted of white drifting horizontal bars (2° wide). The distance between two bars was 70° and they were presented at a temporal frequency of 0.125Hz. To calculate ocular dominance, the visual stimulus was restricted to the binocular visual field of the left V1 (−5° to +15° azimuth, 0° azimuth corresponding to frontal direction) and animals were stimulated through either left or right eye in alternation. To visualize retinotopic maps the stimulus monitor was placed in the right visual field of the animal at a distance of 25cm to optimally stimulate the right eye (contralateral to the recorded hemisphere), while the left eye was covered. In this case, the visual stimuli consisted of a drifting bar extending across the full width of the screen (78° azimuth and 59° elevation, respectively). Both vertical (90° and 270°; elevation orientation) and horizontal (0°and 180°; azimuth orientation) drifting bars were presented.

#### Data analysis

Visual cortical maps were calculated from the acquired frames by Fourier analysis to extract the signal at the stimulation frequency using custom software [29, 30]. While the phase component of the signal is used for the calculation of retinotopy, the amplitude component represents the intensity of neuronal activation (expressed as fractional change in reflectance ×10^−4^) was used to calculate OD. At least 3 maps per animal were averaged to compute the ocular dominance index (ODI) as follows: (C−I)/(C+I), with C and I representing the response magnitudes of each pixel to visual stimulation of the contralateral and ipsilateral eye. The ODI ranges from −1 to +1, with negative values representing ipsilateral dominance and positive values representing contralateral dominance. To determine monocular V1-activation and the quality of the retinotopic maps, we used the calculation introduced by Cang et al., 2005 [31]. For quantification, the most responsive 20,000 pixels in V1 for both azimuth and elevation maps were selected. For every pixel, the difference between its phase and the mean phase of its 24 surrounding pixels was calculated. The standard deviation of the position difference was used as an index of the quality of the retinotopic maps. Lower values indicate lower map scatter and thus higher retinotopic map quality and vice versa.

### Statistical analyses

All intra- and intergroup comparisons were analyzed either by a two-tailed t-test or one-way ANOVA followed by multiple comparisons Bonferroni correction. The intergroup comparison of the enhancement of the spatial frequency and contrast sensitivity thresholds were analyzed by two-way ANOVA. Normal distribution of data was checked using the Shapiro-Wilk test. The levels of significance were set as *p<0.05; **p<0.01; ***p<0.001. Data are represented as means±SEM.

## Results

### Ocular dominance shifts in enriched environment-mice happen already after 2 days of monocular deprivation

It was previously shown that enriched environment (EE) mice exhibit lifelong ocular dominance (OD) plasticity after 7 days of monocular deprivation (MD) [8, 9]. Here we tested whether shorter MD-periods were already sufficient to induce OD-shifts in EE-mice compared to standard cage (SC) mice. Using optical imaging of intrinsic signals, we visualized V1-activation after stimulation of the left and right eye before and after MD. To analyze whether there are age-dependent changes in the speed of OD-shifts, we analyzed EE-mice of three different age groups: critical period mice (postnatal days (P) 24–35), young adult mice (P90-104) and adult mice (P117-283).

**Fig 1:**
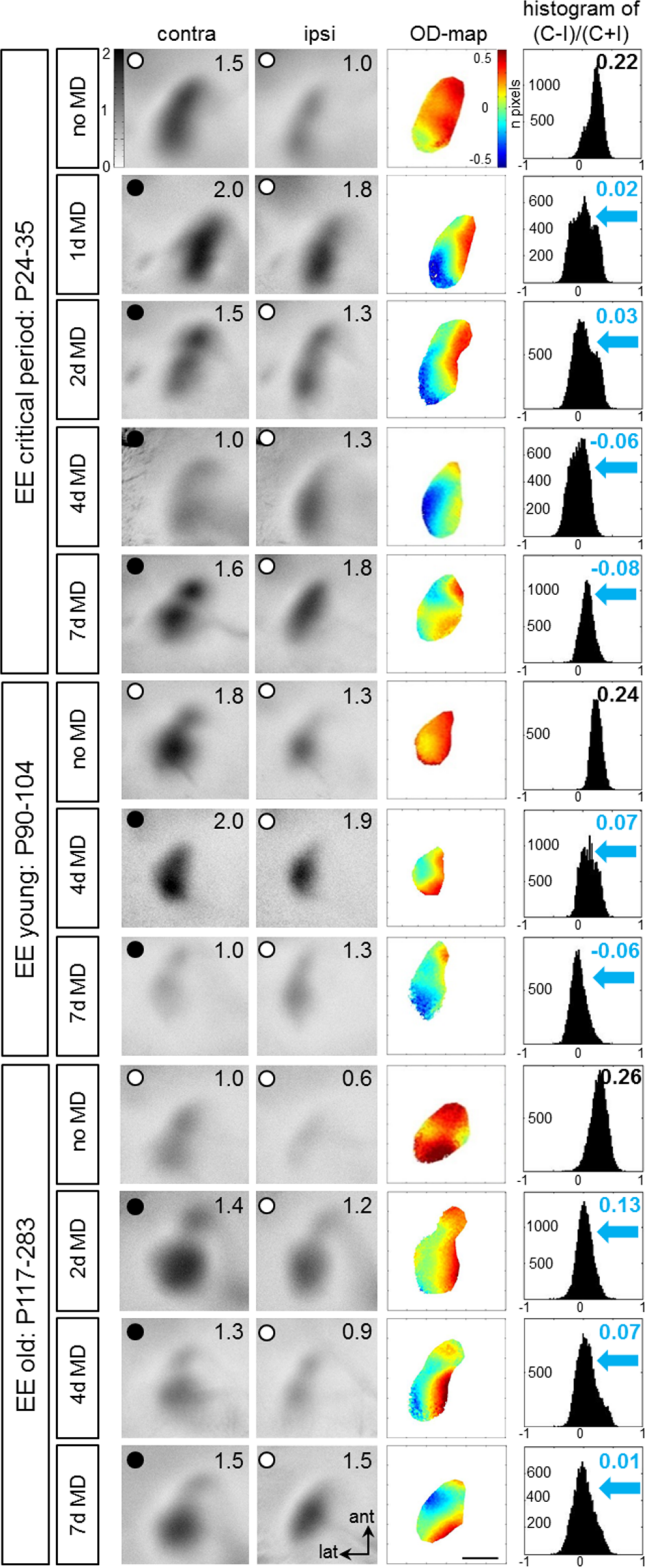
Accelerated ocular dominance (OD) shifts in V1 of enriched environment (EE) mice. Representative examples of optically recorded activity maps in the binocular region of V1 of animals of three different age groups: critical period (P24-35), young (P90-104) and old EE-mice (P117-283), before and after 1, 2, 4 or 7 days (d) of monocular deprivation (MD). Grayscale-coded V1-activity maps (neuronal activation expressed as fractional change in reflectance ×10^−4^) after contralateral (contra) and ipsilateral (ipsi) eye stimulation (numbers correspond to quantified V1-activation of the illustrated example), color-coded 2-dimensional OD-maps and the histogram of OD-scores, including the average ocular dominance index (ODI) are illustrated. Without MD, activity patches evoked by stimulation of the contralateral eye were darker than those of the ipsilateral eye, the average ODI was positive, and warm colors prevailed in the OD-maps, indicating contralateral dominance. MD of 1–2 days already induced strong OD-shifts towards the open eye: after MD, contra- and ipsilateral eye stimulation activated V1 about equally strong, colder colors appeared in the OD-map, and the histogram of OD-scores shifted to the left (blue arrows). Scale bar: 1 mm.

In critical period EE-mice, V1 activation was visualized after 1, 2, 4 or 7 days of MD; age-matched animals without MD served as controls (Fig 1, uppermost 5 rows). In all mice without MD, binocular V1 was dominated by the contralateral eye, average ODIs were positive, and warm colors prevailed in the 2-dimensional OD-maps. After MD, activation in binocular V1 changed: V1 became activated more equally strong by both eyes or became even dominated by the open (previously weaker) ipsilateral eye, ODI values decreased, colder colors appeared in the two-dimensional OD-maps and the ODI-histograms shifted to the left. Notably, experience-dependent changes in V1-activation were already visible after only one day (Fig 1, 2^nd^ row).

Young adult EE-mice (P90-P104) were tested for OD-plasticity after 4 or 7 days of MD (Fig 1, middle three rows). Importantly, at this age, 7 days of MD are necessary to induce an OD-shift in SC-raised mice. In contrast, in EE-mice, OD-shifts were visible already after 4 days of MD. Confirming previous results, also 7 days of MD induced significant OD-shifts in EE-mice, but V1-activation seemed to be generally lower than after 4 days of MD. As expected, V1 of young adult EE-mice without MD was dominated by the contralateral eye.

Finally, a group of even older adult EE-mice (P117-283) was tested for OD-plasticity after 2, 4 or 7 days of MD (Fig 1, lowermost 4 rows). At this age, SC-mice did not reliably show OD-plasticity even after 14 days of MD [19]. As in the younger EE-mice, OD-plasticity was accelerated also in this age group of EE-mice: even 2 days of MD were sufficient to induce significant OD-shifts. Visual stimulation of both contralateral and ipsilateral eye activated V1 similarly strong than without MD, ODI-values were closer to zero, colder colors dominated in the OD-maps and the ODI-histograms shifted to the left. As expected, V1 of control mice without MD remained dominated by the contralateral eye: ODIs were positive and warm colors prevailed in the OD-maps. In summary, in all EE-mice, optically imaged MD-induced V1-activation changes happened much faster than in SC-mice.

**Fig 2:**
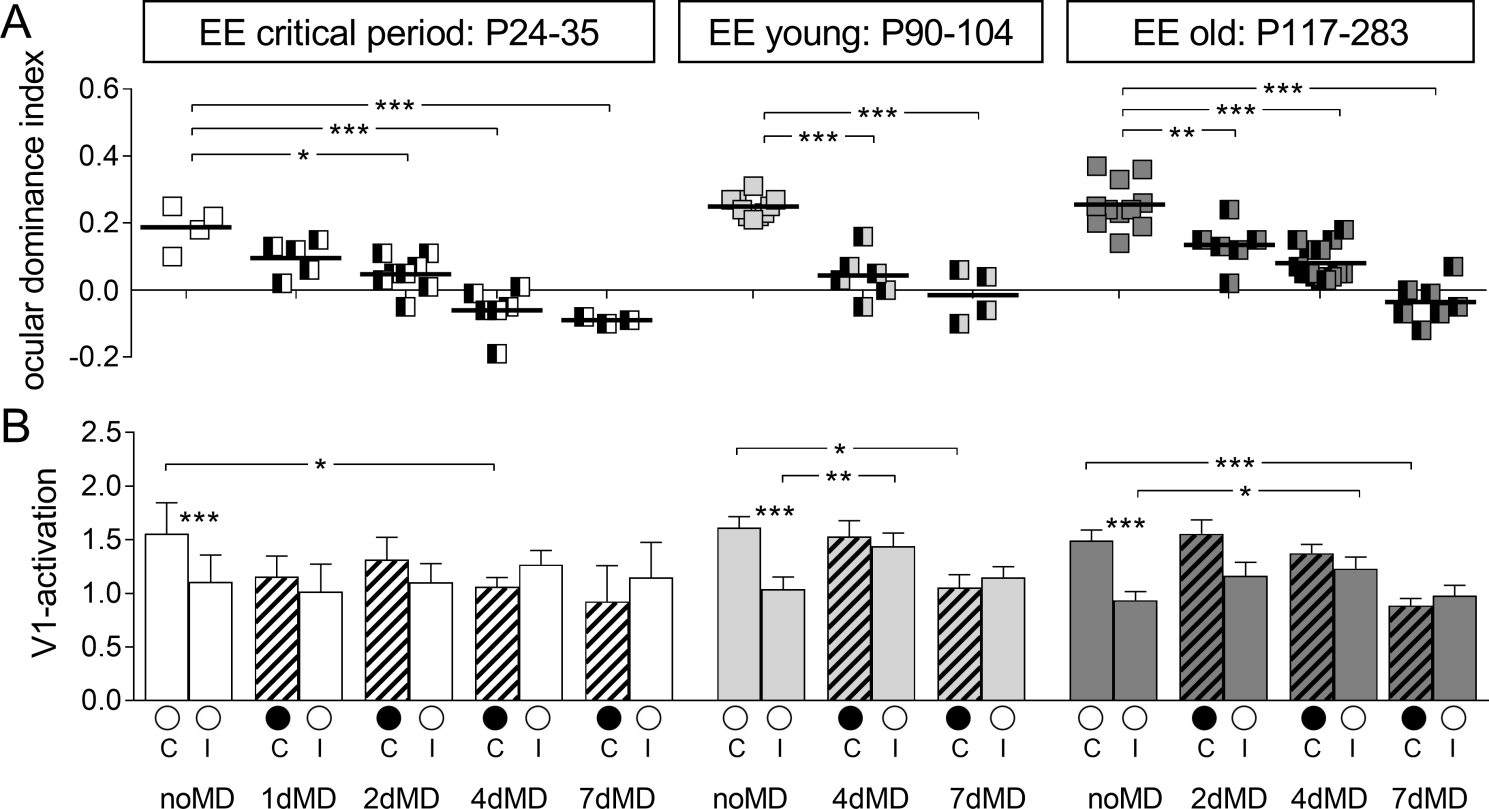
Quantification of optically recorded V1-activation in enriched environment-mice of three different age groups before and after varying monocular deprivation durations. **A.** Optically imaged ODIs of critical period, young and old enriched environment (EE) mice. Symbols represent individual ODI-values, means are marked by horizontal lines; values after monocular deprivation (MD) are indicated by half-black squares. **B.** V1-activation elicited by stimulation of the contralateral (C) or ipsilateral (I) eye without and with MD (black filled circle indicates MD eye).

Quantification of V1-activation of all recorded maps confirmed these observations and clearly showed that EE-mice of all age groups showed a significant OD-shift already after 2–4 days of MD, and thus much faster than what was previously described for SC-mice of similar age groups (Fig 2, upper row). In particular, the ODI of critical period EE-mice shifted already after 2 days of MD from 0.19±0.03 (n = 4, noMD) to 0.06±0.02 (n = 8, P24-31, p = 0.002, Bonferroni-adjusted (B) t-test). The OD-shift after 1 day of MD to 0.10±0.02 (n = 5) was not yet significant (p = 0.054, B t-test). After 4 and 7 days of MD, OD-shifts were also significant: ODI of -0.06±0.03 after 4 days of MD (n = 6, P27-35, p = 0.0005, B t-test) and -0.09±0.03 after 7 days of MD (n = 6, P30-35, p = 0.0008, B t-test).

Young adult EE-mice also displayed significant decreases of the ODI from 0.25±0.01 in animals without MD (n = 10, P90-104) to 0.04±0.03 after 4 days of MD (n = 6, P96-103; p<0.0001, B t-test) and to -0.02±0.04 after 7 days of MD (n = 4, P91-104; p<0.0001, B t-test). Similarly, the OD-shifts were also already significant after only 2 days of MD in old EE-mice: from an ODI of 0.26±0.02 without MD (n = 11; P117-281) to 0.13±0.03 after 2 days of MD (n = 5, P145-283, p = 0.004, B t-test), to 0.08±0.01 after 4 days of MD (n = 14, P119-242, p<0.0001, B t-test) and to -0.04±0.02 after 7 days of MD (n = 7, >P110; p<0.0001, B t-test).

Comparison of V1-activation after visual stimulation of the contra- and ipsilateral eye before and after various periods of MD revealed the following underlying mechanisms of the OD-shifts (Fig 2, lower row). In critical period EE-mice, the OD-shifts seem to be exclusively mediated by reductions in deprived (contralateral) eye responses in V1, because V1-activition after open eye (ipsilateral) stimulation remained unchanged after 2, 4 and 7 days of MD. In detail, in critical period EE-mice without MD (noMD), average V1-activation after contralateral eye stimulation was 1.56±0.29 compared to 1.11±0.25 (n = 4) after ipsilateral eye stimulation. After 1 day of MD, V1-activation after contralateral eye stimulation was already reduced to 1.16±0.19 (n = 5; p = 0.268, B t-test, compared to the noMD group), while ipsilateral eye V1-activation was 1.02±0.26, not significantly different from the noMD mice (p = 0.809, B t-test). After 2 days of MD, V1-activation via the contralateral eye was 1.31±0.21 (n = 8, p = 0.511, B t-test) and 1.10±0.17 after ipsilateral eye stimulation (n = 8, p = 0.987, B t-test, compared to noMD group). After 4 days of MD, V1 activation via the deprived (contralateral) eye was 1.06±0.09 (n = 6, p = 0.037, B t-test), and thus significantly decreased compared to the noMD EE-mice. In contrast, open (ipsilateral) eye V1-responses did not change (1.27±0.13, n = 6, p = 0.55, B t-test). Similarly, after 7 days of MD, deprived/open eye V1-activation was 0.92±0.34/1.15±0.33 (n = 3/3, p = 0.21/0.926, B t-test). Thus, in critical period EE-mice, 2 days of MD were already sufficient to induce a significant OD-shift, and this experience-dependent change of V1-activation seemed to be mediated primarily via a reduction of deprived (contralateral) eye responses in binocular V1.

In young adult EE-mice, OD-shifts were also induced faster than in SC-mice. Interestingly, the OD-shift after 4 days of MD seemed to be mediated via a fast increase in open (ipsilateral) eye responses in V1 followed by a more delayed reduction in deprived eye responses in V1 after 7 days of MD. Already after 4 days of MD, V1-activation after open (ipsilateral) eye stimulation increased from 1.04±0.11 (n = 10; noMD) to 1.44±0.18 (n = 6, p = 0.038, B t-test). In contrast, deprived (contralateral) eye responses in V1 were not different before and after 4 days of MD (V1-activation contra noMD/4dMD: 1.61±0.10 (n = 10)/ 1.53±0.15 (n = 6), p = 0.635, B t-test). Interestingly, the strong OD-shift after 7 days of MD was primarily mediated by a reduction of deprived (contralateral) eye responses in V1 (V1-activation contra noMD/7dMD 1.61±0.10 (n = 10)/ 1.05±0.12 (n = 4), p = 0.008, B t-test), while V1-activation after open (ipsilateral) eye stimulation was not significantly different from the noMD-condition (ipsi noMD/7dMD: 1.04±0.11 (n = 10) /1.15±0.10 (n = 4), p = 0.574, B t-test).

Detailed quantification of activity maps in old EE-mice revealed essentially similar changes of V1-activation after MD. Already after 4 days of MD, V1-activation via the open (ipsilateral) eye was significantly increased (ipsi noMD/4dMD: 0.94±0.08 (n = 11) / 1.18±0.17 (n = 14), p = 0.047, B t-test), while deprived (contralateral) eye responses in V1 were not different from the noMD group (contra noMD/4dMD: 1.49±0.10 (n = 11) / 1.34±0.14 (n = 14), p = 0.393, B t-test). The OD-shift after 7 days of MD was primarily mediated by a reduction in deprived eye responses in V1 (contra noMD/7dMD: 1.49±0.10 (n = 11) / 0.89±0.07 (n = 7), p = 0.0003, B t-test), while open (ipsilateral) eye V1-reponses were not different from the noMD-condition (ipsi noMD/7dMD: 0.94±0.08 (n = 11) / 0.98±0.10 (n = 7), p = 0.737, Bonferroni-adjusted t-test).

**Fig 3:**
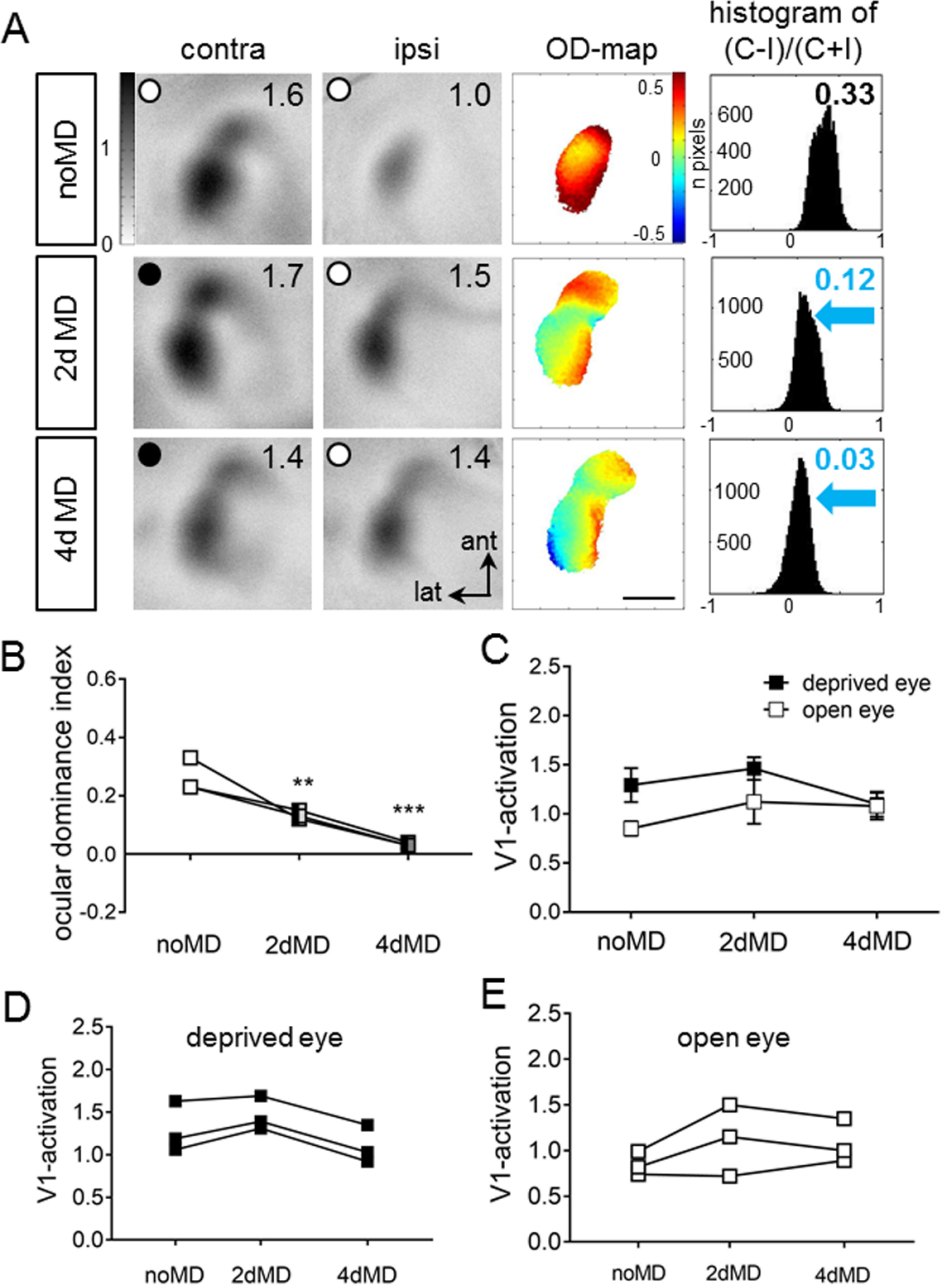
Ocular dominance-shifts happen already after 2 days of monocular deprivation in adult enriched environment mice. Chronic imaging of V1-activation in enriched environment (EE)-mice before and after monocular deprivation (MD), and its quantification. **A**: V1-activity maps of a P143 EE-mouse after visual stimulation of the contra- (contra) and ipsilateral (ipsi) eye, before (noMD, first row) and after 2/4 days of MD (second/third row). Layout and data display as in Fig 1. **B-E**: Ocular dominance indices (B), average V1-activation (C) and individual V1-activation after deprived and open eye stimulation before and after MD of the three chronically imaged EE-mice.

In order to monitor how V1-activity changed after MD, and whether changes were mediated more by a reduction of deprived eye responses or an increase in open eye responses in V1, we additionally performed chronic imaging of V1-activity before and after 2 and 4 days of MD in three adult EE-mice (P143, P218 and P271) (Fig 3). In detail, mice were first imaged directly before MD, then the right eye (contralateral to the imaged hemisphere) was deprived for 2 days and V1 was imaged again (second session). Immediately after the second imaging session the previously deprived eye was again closed for 2 more days and then a third imaging session was performed. As expected, V1-activity maps recorded before MD (first session) were dominated by input from the contralateral eye, warm colors prevailed in the 2-dimensional OD-map and the average ODI was positive (Fig 3A, 1^st^ row). Already after 2 days of MD (second imaging session), a change in V1-activation was observed: now the ipsilateral eye activated V1 more strongly, resulting in decreased ODI-values, colder colors appeared in the 2-dimensional OD-map, and the ODI-histogram shifted to the left (Fig 3A, 2^nd^ row). After 4 days of MD (third session), V1-activation was rather equally activated by both eyes, even more regions of the OD-map were dominated by colder colors, ODI-values were even more reduced and the ODI-histogram was shifted further to the left (Fig 3A, 3^rd^ row). Quantitative analyses of V1-activation showed that the ODI steadily decreased from 0.26±0.03 before MD to 0.12±0.02 after 2 days of MD and to 0.03±0.01 after 4 days MD (n = 3; Fig 3B). Before MD, V1-activation was more strongly activated after contralateral eye compared to ipsilateral eye stimulation (Fig 3C–3E). V1-activation via the open (ipsilateral) eye increased slightly after only 2 days of MD, whereas V1-activation via the deprived (contralateral) eye showed a tendency of a delayed decrease starting after about 4 days of MD.

Taken together, our data demonstrate i) that 2 days of MD were already sufficient to induce significant OD-shifts in EE-mice of all tested age groups, ii) that OD-shifts of critical period mice after 4d MD were primarily mediated by reductions in deprived eye responses in V1, while iii) OD-shifts of older EE-mice were initially mediated by fast increases in open eye responses in V1 (after 2–4 days of MD), followed by a delayed decrease in deprived and open eye responses in V1 (after 4–7 days of MD).

### Basic visual abilities and enhanced optomotor thresholds after monocular deprivation did not change with age in enriched environment -mice

Using the optomotor setup, we additionally determined the spatial frequency and contrast sensitivity thresholds of the optomotor reflex in all EE-mice of the three different age groups. Baseline spatial frequency threshold was 0.38±0.001 cyc/deg (n = 26; P24-35) in critical period EE-mice, 0.38±0.001 cyc/deg (n = 20; P90-104) in young adult EE-mice, and 0.38±0.01 cyc/deg (n = 31; P117-283) in old EE-mice (Table 1). There were no significant differences between the three tested groups (p>0.05, ANOVA).

**Table 1:** Spatial frequency thresholds over the days for each EE-group.

		Day 0	Day 1	Day 2	Day 3	Day 4	Day 5	Day 6	Day 7
**EE critical period**	**noMD**	0.38±0.001	0.38±0.001	0.38±0.001	0.38±0.001	0.38±0.001	0.38±0.001	0.38±0.001	0.38±0.001
	**1dMD**	0.38±0.001	0.39±0.001						
	**2dMD**	0.38±0.001	0.39±0.001	0.4±0.001					
	**4dMD**	0.38±0.001	0.39±0.002	0.4±0.002	0.41±0.002	0.42±0.002			
	**7dMD**	0.38±0.003	0.39±0.004	0.4±0.005	0.41±0.004	0.42±0.004	0.43±0.005	0.44±0.004	0.45±0.004
**EE young**	**noMD**	0.38±0.002	0.038±0.002	0.38±0.002	0.38±0.002	0.38±0.002	0.38±0.003	0.038±0.003	0.38±0.003
	**4dMD**	0.038±0.002	0.39±0.003	0.4±0.003	0.41±0.003	0.42±0.002			
	**7dMD**	0.37±0.008	0.39±0.008	0.41±0.008	0.42±0.02	0.43±0.006	0.44±0.006	0.45±0.006	0.45±0.006
**EE old**	**noMD**	0.38±0.001	0.38±0.001	0.38±0.001	0.38±0.001	0.38±0.001			
	**2dMD**	0.38±0.001	0.39±0.001	0.4±0.001					
	**4dMD**	0.38±0.001	0.4±0.001	0.41±0.002	0.42±0.005	0.43±0.006			

We also determined baseline contrast sensitivity thresholds for all mice (Table 2). There were no significant differences between baseline contrast sensitivities of all groups (p>0.05 for every spatial frequency, ANOVA). Both baseline optomotor thresholds for spatial frequency and contrast were similar to values previously published for SC-raised C57BL/6J mice [32, 33] and old EE-mice [8]. Altogether our measurements demonstrate that baseline visual abilities were similar in all tested age groups of EE-mice, as expected from previous observations [9].

**Table 2:** Contrast sensitivity thresholds before and after monocular deprivation period.

		**EE critical period**				
		**noMD**	**1dMD**		**2dMD**	
			baseline	day 1	Baseline	day 2
**Spatial frequency**	**0.031**	3.7±0.04	3.7±0.01	3.8±0.01	3.7±0.02	3.9±0.02
	**0.064**	13.2±0.21	12.5±0.28	13.4±0.34	12.5±0.16	14.5±0.13
	**0.092**	12.2±0.06	11.8±0.23	12.6±0.24	11.7±0.13	13.3±0.11
	**0.103**	11.5±0.08	11.2±0.21	12.0±0.20	11.2±0.12	12.6±0.15
	**0.192**	7.6±0.09	7.6±0.09	8.0±0.08	7.8±0.07	8.7±0.08
	**0.272**	3.7±0.04	3.6±0.01	3.7±0.02	3.6±0.01	3.8±0.02
			**4dMD**		**7dMD**	
			baseline	day 4	Baseline	day 7
**Spatial frequency**	**0.031**		3.7±0.02	4.3±0.04	3.8±0.06	4.6±0.15
	**0.064**		13.5±0.14	20.9±0.34	13.0±0.17	24.6±0.52
	**0.092**		12.3±0.16	18.0±0.35	12.9±0.28	21.5±0.31
	**0.103**		11.7±0.15	16.7±0.24	11.7±0.2	19.8±0.34
	**0.192**		7.5±0.06	8.9±0.98	7.5±0.05	11.4±0.15
	**0.272**		3.7±0.02	4.1±0.03	3.7±0.01	4.5±0.02
		**EE young**				
		**noMD**	**4dMD**		**7dMD**	
			baseline	day 4	Baseline	day 7
**Spatial frequency**	**0.031**	3.6±0.22	3.7±0.02	4.0±0.04	3.9±0.12	5.5±0.32
	**0.064**	11.5±0.24	12.1±0.24	16.6±0.47	12.4±0.49	28.4±3.6
	**0.092**	11.3±0.22	11.6±0.23	15.9±0.45	11.4±0.47	23.9±2.75
	**0.103**	11.0±0.27	11.1±0.27	15.2±0.44	10.7±0.49	20.7±2.15
	**0.192**	7.5±0.19	7.6±0.24	9.9±0.37	7.9±0.13	13.2±0.7
	**0.272**	3.6±0.04	3.7±0.04	4.1±0.04	4.0±0.16	5.9±0.34
		**EE old**				
		**noMD**	**2dMD**		**4dMD**	
			baseline	day 2	Baseline	day 4
**Spatial frequency**	**0.031**	3.8±0.03	3.7±0.01	3.9±0.01	3.9±0.04	4.7±0.09
	**0.064**	12.1±0.20	12.6±0.14	14.5±0.12	12.3±0.21	19.8±1.22
	**0.092**	11.4±0.06	11.7±0.21	13.4±0.16	11.6±0.18	17.9±1.05
	**0.103**	10.9±0.11	11.0±0.28	12.4±0.23	11.2±0.13	16.9±0.79
	**0.192**	7.8±0.15	7.5±0.15	8.5±0.19	8.0±0.20	10.7±1.08
	**0.272**	3.7±0.02	3.6±0.03	3.7±0.02	3.7±0.03	4.9±0.44

**Fig 4:**
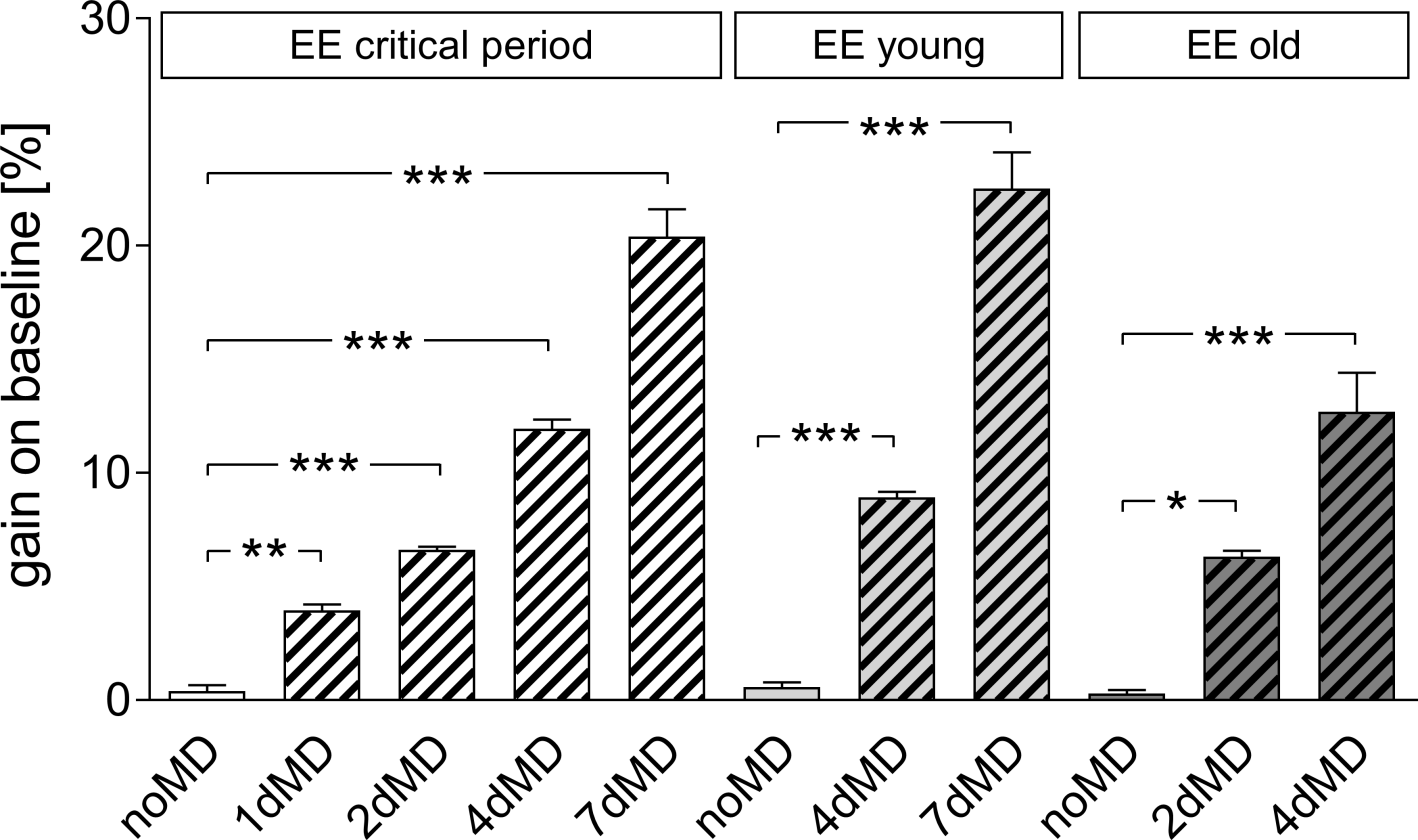
Increase of optomotor thresholds of the open eye after monocular deprivation in enriched environment -mice. Gain on baseline [%] of spatial frequency thresholds of the optomotor reflex, determined by optomotry, plotted for the three different age groups of EE-mice and separated for the various MD-durations.

Next, we measured the experience-induced improvements in spatial frequency and contrast sensitivity thresholds of the optomotor reflex of the open eye after MD and daily training in the optomotor setup. All three age groups of EE-mice showed a significant increase in spatial frequency (Fig 4; Table 1) and contrast sensitivity thresholds of the optomotor reflex over the MD-period. Specifically, in critical period mice, spatial frequency thresholds increased by 3.9±0.3% after 1 day of MD (n = 5), by 6.6±0.2% after 2 days of MD (n = 8), by 12.0±0.4% after 4 days of MD (n = 6) and by 15.2±0.3% after 7 days of MD (n = 3). All increases were significantly different from values before MD (p<0.001, B t-test for all comparisons). In young EE-mice, the increase in spatial frequency threshold was 8.9±0.24% (n = 6) after 4 days of MD and 9.0±0.16% (n = 4) after 7 days of MD. Again, all increases were significantly different from values before MD (p<0.001, B t-test for all comparisons). Spatial frequency thresholds increased similarly after MD for the old EE-mice: by 6.3±0.27% (n = 6) in the 2dMD group and by 10.3±0.73% (n = 4) in 4dMD group (p<0.001, B t-test, for all comparisons with the noMD group). Mice without MD from all age groups did not show improvement in spatial frequency threshold over the days (p>0.05, compared to day 0, Bonferroni-adjusted t-test; Table 1).

The contrast sensitivity thresholds of the optomotor reflex of the open eye also increased over the MD-period in mice of all tested age groups (Table 2). Values of animals without MD did not change over days. Contrast sensitivity thresholds were not different between the groups on each day after MD (p>0.05 for every frequency, 2-way ANOVA, Table 2).

To conclude, neither basic visual abilities nor the experience-enabled increase of spatial frequency and contrast sensitivity thresholds after MD were different in the three tested groups of EE-mice.

### Transfer of enriched environment mice to standard cages immediately abolished ocular dominance plasticity

**Fig 5:**
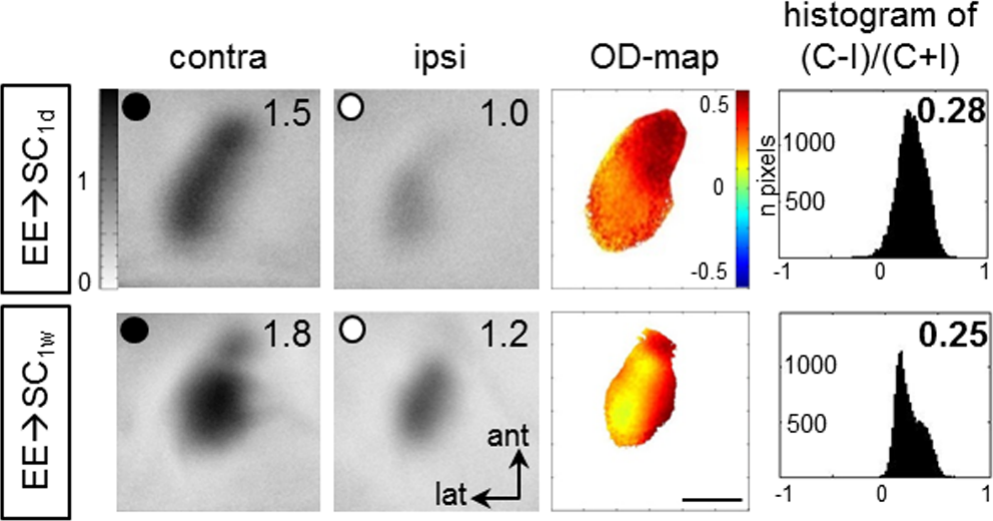
Transferring enriched environment-mice to standard cages (SCs) immediately abolished ocular dominance-plasticity. Intrinsic signal imaging of V1-activation in EE→SC-mice after MD, and its quantification. MD was induced after 1 day (1d) or one week (1w) in SC. Layout and data display as in Fig 1.

Enriched environment (EE) housing has not only been shown to extend the sensitive phase for ocular dominance (OD) plasticity into late adulthood but could also restore OD-plasticity to adult standard cage (SC)-raised mice [8]. Here we tested how long the plasticity promoting effect of EE-housing would persist after transferring EE-mice back to the rather deprived environment of a SC. To this end, mice born and raised in EE-cages until at least P130 were transferred to SCs (EE→SC-mice), and received a 7-day-monocular deprivation (MD) after either 1 d or 1 wk in SC to test OD-plasticity. After the end of the MD-period, V1-activation of all animals was optically imaged. Surprisingly, even after MD, the binocular part of V1 of both EE→SC-mice groups (MD after 1 day or one week) remained dominated by the deprived, contralateral eye: activity patches induced by visual stimulation of the deprived eye were darker than those after stimulation of the open (ipsilateral) eye, ODIs were positive and warm color dominated the 2-dimensioned OD-maps (Fig 5).

Quantitative analysis of the ODIs showed that OD-plasticity was absent in both groups of EE-mice transferred to SC either 1 week (EE→SC_1w_) or one day before MD (EE→SC_1d_). Specifically, the average ODI of EE→SC_1w_–mice was 0.23±0.01 (n = 4, P162-281), and 0.23±0.03 (n = 4, P164-278) for EE→SC_1d_-mice. The difference between the two groups was not significant (p = 0.759, t-test). However, comparison of these ODIs to values of age matched EE-mice (8) after MD revealed a significant difference (p<0.0001, for both groups, ANOVA) but not when compared to values of age-matched SC-mice after MD (p = 0.525 and p = 0.426 for EE→SC_1w_ and EE→SC_1d_-mice, respectively; B t-test; SC-values from Lehmann and Löwel, 2008). Thus, EE→SC mice reacted to an MD like SC-mice of the same age. Further quantitative analyses of V1-activation showed a clear contralateral dominance in both EE→SC groups. In EE→SC_1w_-mice, V1-activation after contralateral eye stimulation was 1.54±0.24 and 1.08±1.18 after ipsilateral eye stimulation. Similarly, in EE→SC_1d_ -mice, V1-activation after contralateral/ipilateral eye stimulation was 1.92±0.07/1.28±0.08. Taken together, our data indicate that already after one week in SC OD-plasticity, which is otherwise preserved in EE-mice, is lost.

### Basic visual abilities and improvements of the optomotor reflex in enriched environment mice transferred to standard cages after monocular deprivation were similar as in enriched environment and standard cage mice

Using optomotry, spatial vision (spatial frequency and contrast thresholds) of all enriched environment mice transferred to standard cage (EE→SC) mice was also measured. Baseline spatial frequency threshold of the optomotor reflex was 0.38±0.004 cyc/deg while the average contrast sensitivity threshold for the 6 tested spatial frequencies were 3.9±0.04/13.7±0.26/12.9±0.24/11.8±0.19/8.4±0.34/3.8±0.04 for 0.031/0.064/0.092/0.103/0.192/0.272 cyc/deg. These values were similar to values published for age-matched SC- and EE-mice [8, 33].

After 7 days of MD, the spatial frequency threshold of the open eye increased to 0.44±0.002 cyc/deg (p<0.001 compared to day 0, B t-test). Likewise, contrast sensitivity thresholds increased after MD: to 4.8±0.07/25.1±1.4/21.4±0.7/19.5±1.4/11.9±0.8/4.5±0.1 at 0.031/0.064/0.092/0.103/1.192 and 0.272 cyc/deg (p>0.05, p<0.001, p<0.001, p<0.001, p<0.01, p>0.05, compared to baseline values on day 0, ANOVA). Experience-dependent threshold increases for both spatial frequency and contrast sensitivity were not different from previously published values of age-matched SC- and EE-mice after MD [8, 32].

### OD-plasticity was rescued in EE→SC mice by adding a running wheel to standard cages, but not by treatment with the selective serotonin reuptake inhibitor fluoxetine

**Fig 6:**
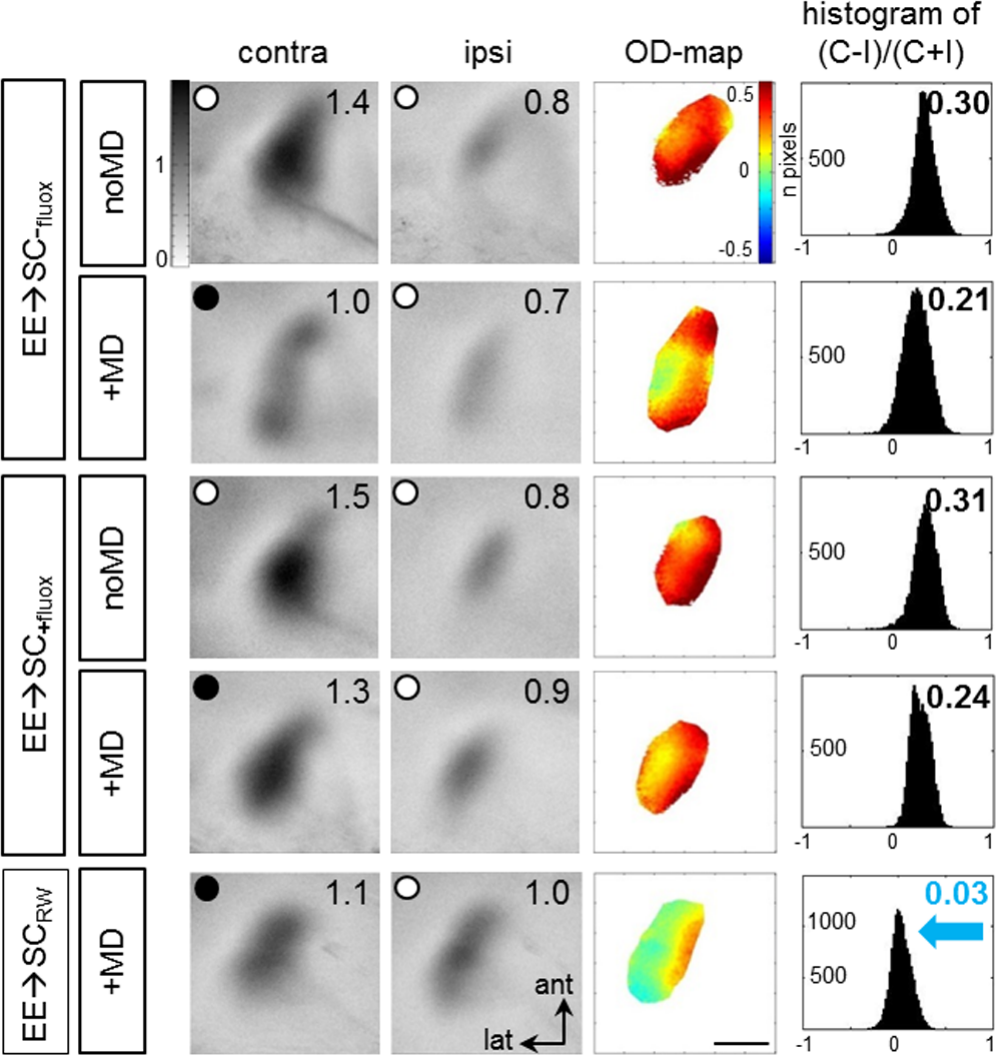
Adding a running wheel to standard cages but not fluoxetine treatment rescued ocular dominance plasticity after transferring EE-mice to SCs (EE→SC). Examples of V1-activity maps and their quantification recorded from EE-mice transferred to SCs: EE->SC-mice without treatment (upper 2 rows), with fluoxetine treatment (3^rd^ and 4^th^ row), or with added RW in the SCs (5^th^ row). Layout and data display as in Fig 1. MD only induced an OD-shift in V1 of the RW-group (EE→SC_RW_): after MD, the contra- and ipsilateral eye activated V1 about equally strong, colder colors appeared in the OD-map, and the histogram of OD-scores shifted to the left (blue arrow). In all other groups, including those treated with fluoxetine, V1 remained dominated by input from the contralateral (deprived) eye: contralateral eye evoked activity patches were darker than those of the ipsilateral eye, the average ODI was positive, and warm colors prevailed in the OD-maps. Scale bar: 1 mm.

It had been shown previously that fluoxetine restored OD-plasticity in adult rats [24]. To test whether fluoxetine would also prevent the disappearance of OD-plasticity in EE→SC mice, we supplemented the drinking water of EE→SC mice immediately after the transfer to SCs with fluoxetine for a total of three weeks (for details of drug application see methods). After 2 weeks of drug (+fluox or control: -fluox) application, we performed MD in half of the animals, and imaged V1-activation 7 days later. Binocular V1 of all 4 groups of mice (EE→SC_-fluox_ noMD/MD and EE→SC_+fluox_ noMD/MD) was dominated by stimulation of the contralateral eye both before and after MD (Fig 6, upper 4 rows): activity patches after contralateral eye stimulation were always darker than those after ipsilateral eye stimulation, warm colors prevailed in the 2-dimensional OD-maps and the average ODIs were positive. Thus adding fluoxetine to the drinking water of EE→SC mice did *not* rescue OD-plasticity.

Since adding a RW to a SC has recently been demonstrated to preserve OD-plasticity in SC-mice into adulthood [28], we here tested whether voluntary physical exercise would rescue OD-plasticity in adult EE→SC mice. This was indeed the case: after MD in EE→SC_RW_ mice, V1 was no longer dominated by the deprived, contralateral eye, but activated about equally strong after contralateral and ipsilateral (open) eye stimulation, colder colors appeared in the OD-map and the ODI-histogram was shifted to the left (Fig 6, lowest row), clearly indicating preserved OD-plasticity.

**Fig 7:**
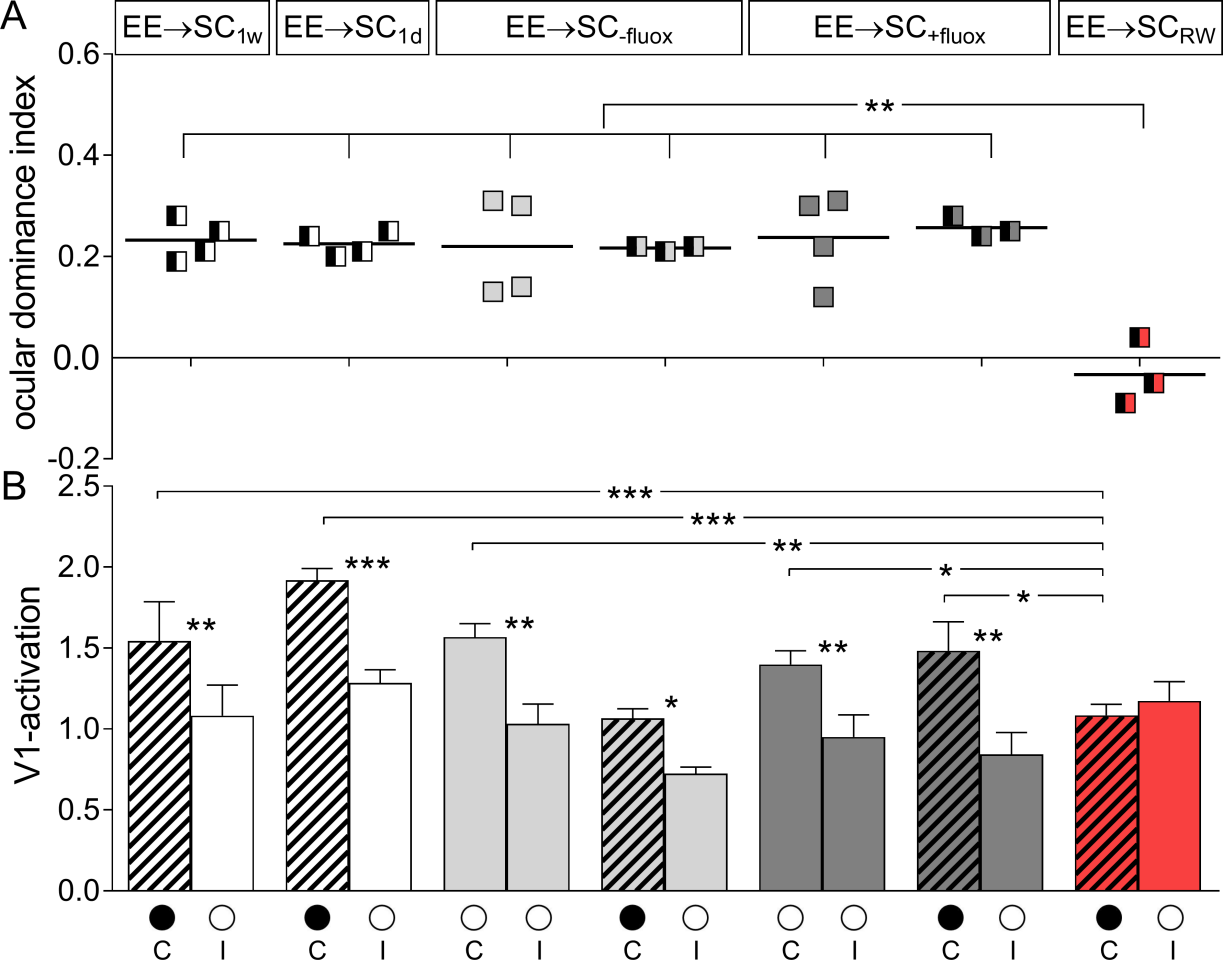
ODIs and V1-activation of EE-mice transferred to standard cages (SCs). **A.** Optically imaged ODIs without and with MD of EE-mice transferred to SC for one week (EE→SC1_w_) or one day (EE→SC1_d_) before MD (white), EE→SC_-fluox_ (light grey), EE→SC_+fluox_ (dark grey) and EE→SC_RW_ mice (red). **B.** V1-activation elicited by stimulation of the contralateral (C) or ipsilateral (I) eye without and after MD. Only in EE→SC_RW_ mice showed an OD-shift after MD: both eyes activated V1 about equally strong whereas in all other groups, V1 continued to be dominated by the previously deprived contralateral eye. Layout and data display as in Fig 3.

Quantification of V1-activation in all EE→SC-mice confirmed these observations and revealed that only the EE-animals transferred to a SC with a RW preserved OD-plasticity (Fig 7, upper row): ODIs of all other groups, including the fluoxetine-treatment group, remained at positive values, even after MD. The average ODI of EE→SC_-fluox_-mice was 0.22±0.05 (n = 4, P266-274) and remained unchanged after MD (0.22±0.01, n = 3, P266-269; p = 0.956, B t-test). EE→SC_+fluox_mice displayed an average ODI of 0.24±0.04 without MD (n = 4, P260-284) and 0.26±0.01 after MD (n = 3, P278-283). Values of mice with and without MD were not different (p = 0.732, B t-test). The ODIs were also not different between mice treated and non-treated with fluoxetine after MD (p = 0.327, B t-test). Only the average ODI for the EE→SC_RW_-mice with MD was significantly different to all other groups (0.21±0.01, n = 3, P211-298; p<0.01, for every group comparison, B t-test).

Comparing V1-responses after contra- and ipsilateral eye stimulation revealed that V1 of all animals except the EE→SC_RW_ mice remained dominated by the contralateral eye, even after MD (Fig 7, lower row). In detail, V1-activity of EE→SC_-fluox_-mice after stimulating the contralateral/ipsilateral eye was 1.57±0.08/0.95±0.08. Values were not different after MD (1.03±0.12/0.72±0.04; MD/noMD comparison: contra-/ipsilateral: p = 0.173/0.443, t-test; contra-/ipsilateral comparison for the MD group: p = 0.026, t-test). Similarly, V1-activation of EE→SC_+fluox_ mice was not different without and with MD, and contra-/ipsilateral eye evoked V1-activation did not change: V1-activation after contra-/ipsilateral eye was 1.40±0.09/0.95±0.14 without MD and 1.48±0.18/0.84±0.14 after MD. Neither of these comparisons were significantly different (p>0.05 ANOVA).

Notably, V1 of mice transferred from EE to a RW-cage (EE→SC_RW_) was activated about equally strong by both eyes after MD (V1 contra/ipsi: 1.08±0.07/1.17±0.66; p = 0.323, t-test). Comparing V1-activation of the EE→SC_RW_ to values of all other groups of EE→SC-mice revealed that contralateral eye evoked activity was significantly lower (p<0.05, for all comparisons, ANOVA) suggesting that the OD-shift of the EE→SC_RW_ mice after 7 days of MD was mediated primarily by a reduction of deprive eye responses in V1.

In conclusion, fluoxetine treatment did not preserve OD-plasticity in EE-mice transferred to SCs, but giving the mice the possibility for voluntary physical exercise by adding a RW did.

#### Fluoxetine treatment did not affect the formation and the strength of V1-maps

**Fig 8:**
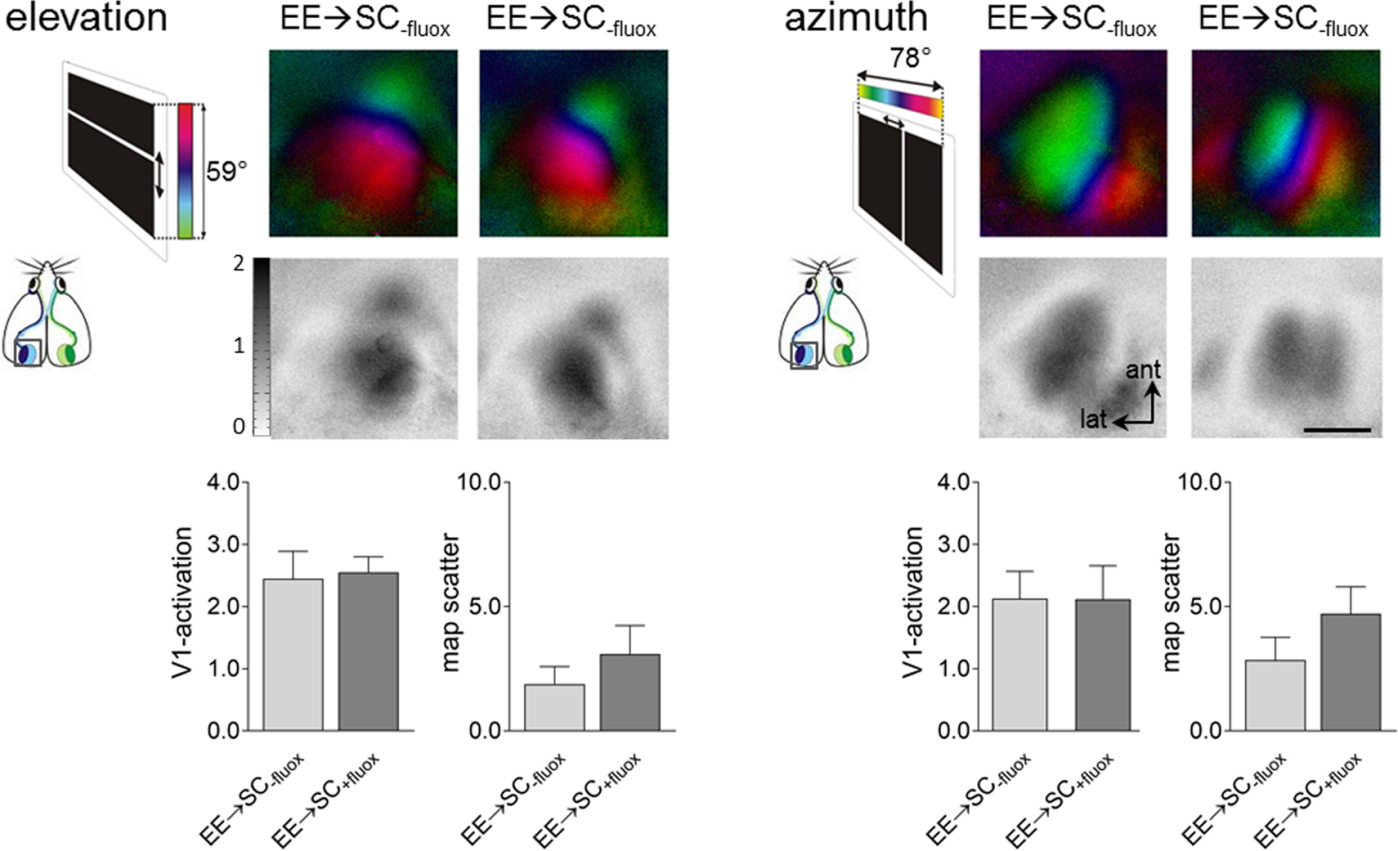
Fluoxetine treatment had no effect on V1-activation nor on the quality of the optically recorded V1-maps. Retinotopic polar and activity maps after visual stimulation with moving horizontal (elevation) or vertical (azimuth) bars (see inset to the left of the V1-maps) recorded from EE→SC mice control (water) or fluoxetine treatment, and their quantification. Comparison of V1-activation (lower left) and map quality (lower right) after contralateral (right) eye stimulation in both control (water, grey) and fluoxetine-treated (red) mice, for both elevation and azimuth stimulation. Recorded V1-maps of both treatment groups were indistinguishable in signal strength and retinotopic map quality. Scale bar: 1 mm.

In order to check whether fluoxetine impacted on the signaling circuits of V1, V1-activation and map quality measurements were additionally performed in water or fluoxetine treated EE-mice after transferring them to SCs. No differences were found concerning the strength of V1-activation, nor the quality of the retinotopic maps between non-treated (water only) or treated mice with fluoxetine (Fig 8). After elevation stimulation, V1-responses of EE→SC_-fluox_-mice were on average 2.45±0.44 and not significantly different from V1-responses of EE→SC_+fluox_-mice which was 2.55±0.25 (p = 0.833, t-test). Likewise, after azimuth stimulation, V1-activation evoked by right eye stimulation was similar between the non-treated and fluoxetine-treated mice (water/fluoxetine: 2.13±0.22/2.12±0.24; p = 0.97, t-test).

Additional quantitative analyses of V1-activation revealed that both elevation and azimuth retinotopic maps were similar in EE→SC_-fluox_-mice and in EE→SC_+fluox_–mice (elevation, -fluox/+fluox: 1.9±0.7/3.1±1.2; p = 0.403, t-test; azimuth, -fluox/+fluox: 2.9±0.9/4.7±1.1; p = 0.232, t-test). Altogether these data suggest that fluoxetine treatment did neither affect V1-activation nor the quality of the imaged retinotopic maps.

### Basic visual abilities and improvements of the optomotor reflex after MD were not affected by fluoxetine treatment or running

Before and during the MD-period, all mice were additionally and daily tested in the optomotor setup [27] to assess spatial frequency and contrast sensitivity thresholds of their optomotor reflex. Baseline spatial frequency values measured on day 0 were not different between treated and non-treated animals. Specifically baseline spatial frequency threshold for EE→SC_-fluox_-mice was 0.373±0.001 cyc/deg (n = 7) and 0.373±0.0002 cyc/deg, (n = 9) for EE→SC_+fluox_ mice. The EE→SC_RW_-mice had a baseline spatial frequency threshold of 0.374±0.0005 cyc/deg (n = 3). The differences among all the groups were not significant (p = 0.884, ANOVA). We also determined contrast sensitivity thresholds of the optomotor reflex for all groups on day 0 (Table 3). Baseline values were similar in all tested mice and for all 6 spatial frequencies tested irrespective of the fluoxetine treatment or RW housing (p>0.05 for every frequency and for every treatment; ANOVA).

**Table 3:** Contrast sensitivity thresholds for each group transferred in SC before and after MD.

		EE➔SC_-fluox_		EE➔SC_+fluox_		EE➔SC_RW_	
		baseline	day 7	baseline	day 7	baseline	day 7
**Spatial frequency**	**0.031**	3.7±0.01	5.2±0.13	3.7±0.002	5.0±0.06	3.7±0.002	5.0±0.13
	**0.064**	13.2±0.14	34.8±0.88	13.3±0.07	48.7±4.12	13.3±0.19	52.6±1.88
	**0.092**	12.6±0.23	32.3±0.66	12.8±0.07	42.8±3.47	12.7±0.20	45.6±1.77
	**0.103**	12.1±0.09	26.9±0.60	12.4±0.02	38.2±2.56	12.0±0.11	41.8±1.18
	**0.192**	7.5±0.01	14.0±0.52	7.4±0.02	14.6±0.77	7.4±0.04	14.5±0.35
	**0.272**	3.7±0.005	5.0±0.13	3.7±0.01	5.0±0.11	3.7±0.01	4.9±0.05

**Fig 9:**
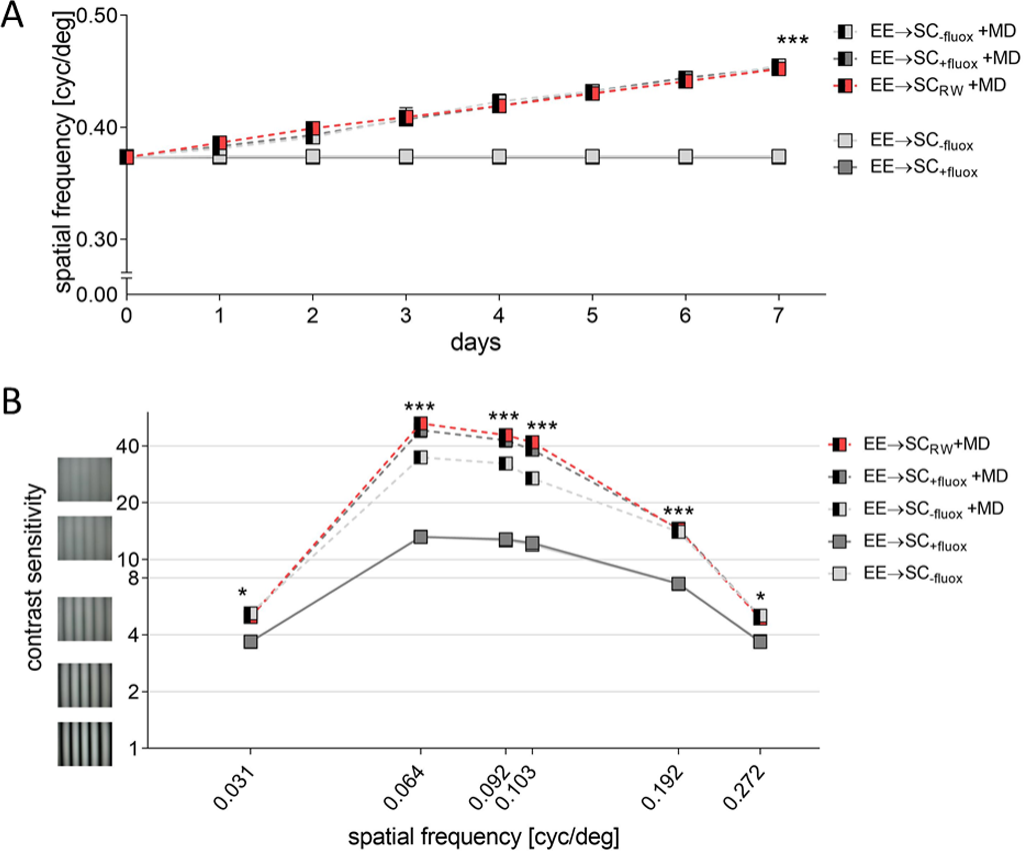
Experience-enabled enhancement of the optomotor reflex of the open eye of enriched mice transferred to a standard cage after monocular deprivation (MD). Both spatial frequency (A) and contrast sensitivity thresholds (B) of the optomotor response before and after MD are compared in control mice, animals treated with fluoxetine or with access to a RW. **A.** Spatial frequency thresholds in cycles per degree (cyc/deg) are plotted against days. Half-filled boxes indicate MD-groups. After 7 days of MD, thresholds improved significantly compared to mice without MD. **B.** Contrast sensitivity thresholds at 6 different spatial frequencies before (day 0) and 7 days after MD of EE→SC_-fluox_, EE→SC_+fluox_ and EE→SC_RW_ mice with or without MD. Threshold values of all groups improved significantly after MD at all tested spatial frequencies compared to the noMD groups (filled boxes).

Experience-dependent changes of spatial frequency thresholds were also measured during the noMD/MD-period for treated and non-treated EE→SC-animals (Fig 9A). As expected, all mice without MD did not improve in spatial frequency thresholds over days (EE→SC_-fluox_/EE→SC_+fluox_ on day 7: 0.374±0.001/0.373±0.001 cyc/deg, n = 4/4; p>0.05 for both groups compared to day 0, ANOVA). In contrast, there was a significant experience-dependent increase in spatial frequency thresholds on day 7 after MD for all EE→SC-groups. The spatial frequency threshold of the open eye of EE→SC_-fluox_-mice increased by 21.8% to 0.455±0.002 cyc/deg on day 7 (n = 3, p = 0.0012, compared to day 0, ANOVA). Similarly, threshold values of EE→SC_+fluox_-mice increase by 21.5% to 0.453±0.003 cyc/deg (n = 3, p = 0.0016, compared to day 0, ANOVA). There was no difference in spatial frequency thresholds on day 7 between the MD groups (treated vs. non-treated: p>0.05, ANOVA). Finally, thresholds of EE→SC_RW_-mice increased by 21.2%, from 0.373±0.005 on day 0 to 0.452±0.001 on day 7 after MD (n = 3, p = 0.0019, ANOVA). Again, increases were not different from the other two groups (EE→SC_-fluox_/EE→SC_+fluox_: p = 0.973/0.856, ANOVA).

Contrast sensitivity thresholds were also determined over the 7 days of MD/noMD period at six different spatial frequencies for all groups (Fig 9B, Table 3). After 7 days of MD, thresholds of both EE→SC_-fluox_- and EE→SC_+fluox_-mice increased compared to mice without MD (p<0.001 for both groups, ANOVA). Also, EE→SC_RW_ mice showed a significant improvement on the 7^th^ day compared to day 0 (p<0.001, ANOVA). The differences among the MD-groups were again not significant (p>0.05, ANOVA). Improvements of optomotor thresholds were similar to previously published data of C57BL/6 mice [33]. Altogether, visual capabilities and their experience-induced changes were not measurably affected by the treatment nor the changes in housing conditions of all EE→SC-mice, in contrast to OD-plasticity in the same animals.

### Fluoxetine treatment did neither change the average water consumption nor the body weight of the mice

Fluoxetine was administered to the mice via the drinking water [25]. To this end, we used dripping free bottles and the water consumption (with or without fluoxetine) was measured daily during the 3 weeks of treatment, and an average daily consumption of the drinking water was calculated.

Mice drinking just water consumed on average 4.01±0.18 ml per day and mice drinking fluoxetine-supplemented water drunk 3.64±0.25 ml per day (difference was not significant: p = 0.237, t-test). For this study, the desired concertation of fluoxetine per mouse (10 mg/kg) [25] was calculated based on the average mouse weight and assuming that the mice drink on average 5 ml of water per day [26]. Thus, the fluoxetine dosage of our mice was 7.28 mg/kg fluoxetine per day, and thus slightly less than attempted.

In addition to the amount of drinking water, the weight of each mouse was measured daily during the 3-week-treatment. Water-only mice had an average weight of 19.8±0.5 g when transferred to SCs; weight increased to 23.7±0.2 g on the last day of treatment. Similarly, fluoxetine-treated mice had an average weight of 23.1±0.3 g when transferred to SC and reached 24.3±0.4 g on the last day of treatment. There was no significant change of weight observed in animals during the SC-housing period (p = 0.078, ANOVA) and both groups had comparable weight on the last day (p = 0.146, t-test).

## Discussion

Neuronal plasticity is crucial for proper development and function of the central nervous system. As the brain matures, its ability to change declines, together with the ability to learn, memorize or recover from various brain injuries. One of the approaches shown to improve brain plasticity is enriched environment (EE). Raising mice in EE was shown to prolong OD-plasticity into adulthood [8, 9]. Additionally, EE-housing restored OD-plasticity in adult mice transferred from standard cages (SCs) to EE when they were older than P110, whereas OD-plasticity was abolished at this age, when mice were housed in SCs [8]. Here we investigated whether experience-dependent shifts of V1-activation happen faster in EE-mice using intrinsic signal optical imaging to visualize V1-activation before and after MD in EE-mice of different age groups. In addition, we investigated what happens to OD-plasticity if EE-mice are transferred to SCs. We observed that already after 2 days of MD, EE-mice displayed a significant OD-shift towards the open eye, much faster than was previously observed in SC-raised mice. In contrast, transfer of EE-mice to SCs immediately abolished OD-plasticity. While fluoxetine-treatment did not restore OD-plasticity in V1 of EE→SC-mice, adding a running wheel (RW) to the SCs preserved OD-plasticity.

The postnatal critical period is a sensitive phase during early life, in which brief alterations in visual experience or neuronal activation can easily and quickly induce cortical plasticity. Given that EE has such a powerful impact on OD-plasticity of adult mice, it was important to test whether EE influences OD-plasticity during the critical period. Indeed, we found that already 2 days of MD were sufficient to induce a clear OD-shift in critical period EE-mice, about twice the speed of age-matched SC-mice. Otherwise, the optically imaged OD-plasticity in young (critical period) EE-mice was similar to previously published data from age-matched SC-mice after 4 days of MD [16–18]. Furthermore, the OD-shift of the young (P24-35) EE-mice after 4 days of MD was comparably strong to the older EE-mice after 7 days of MD [8], and—in both cases—the OD-shift was primarily due to reduced closed eye responses in V1.

OD-plasticity in SC-mice is gradually decreasing as the animals mature. Several manipulations have been proposed to extend the sensitive period for OD-plasticity in adult rodents. Although EE-housing has been proposed as an effective manipulation to promote plasticity in rats [5, 7, 34, 35] and mice [8] it is still unclear whether EE extends the critical period or simply increases the level of adult OD-plasticity. Critical period plasticity is open for a limited duration of time and is quantitatively and qualitatively different from adult OD-plasticity [11]. Characteristics of “juvenile” OD-plasticity in SC-mice are that 4 days of MD are sufficient to induce an OD-shift, and that V1-activation changes are primarily mediated by reductions in deprived eye responses in V1 after 4 days of MD [11]. If the OD-plasticity of old EE-mice is indeed “juvenile-like” then 4 days of MD would be enough to induce a significant OD-shift in these mice. In fact, in both young and old EE-mice, even a 2-day-MD was sufficient to induce a significant OD-shift towards the open eye, clearly showing strongly accelerated changes of V1-activation in EE- compared to SC-mice. While we did not test 2 days of MD in P90-104 EE-mice, also in this age group, OD-shifts of EE-mice were much faster than in age-matched SC-mice (4 vs. 7 days). Our chronic imaging experiments with old EE-mice before and after 2 and 4 days of MD support these conclusions: OD-shifts were already visible after only 2 days of MD and increased with longer MD-duration. Consequently, the surrounding environment had a strong influence on OD-plasticity and mice raised in EE displayed fast changes in cortical activation, even when they were old.

How about the mechanism underlying the accelerated OD-shifts of EE-mice? Previous analyses of OD-plasticity after 7 days of MD in adult EE-mice indicated that OD-shifts were primarily mediated by a reduction of deprived (contralateral) eye responses in V1, indicating that OD-shifts are “juvenile-like”: compared to V1-activation before MD, only changes of deprived eye responses after 7 days of MD were observed, while open eye responses in V1 were not different [8]. Present data provide more details and a higher time resolution of the ongoing V1-activation changes after MD in EE-mice: starting as early as 2–4 days after MD, there is an increase of open eye responses in V1 of old EE-mice (P117-283), followed by a delayed decrease of deprived eye responses after 4–7 days of MD. These V1-activation changes in adult/old EE-mice are thus clearly different from the changes in both young (critical period) SC- and EE-mice. Interestingly, an increase in open eye responses in V1 has so far been only observed after longer deprivation periods; that these changes happen much faster in EE-mice may be taken as an additional indication for accelerated V1-activation changes in less deprived mice, or decelerated changes in SC-mice.

In a recent study, it was shown that a disinhibitory microcircuit initiated OD-plasticity in critical period SC-mice: after an initial drop of evoked firing rates in excitatory V1-neurons, they returned to normal during the first day after MD [36]. This restoration resulted from a fast reduction of excitatory input to PV cells. In this scenario, visual stimulation no longer elicits strong inhibitory responses, resulting in the restoration of normal levels of evoked firing rates in excitatory neurons, in the face of continued MD [36–38]. Thus, and since inhibitory tone of adult EE-mice is as low as in critical period SC-mice [8], it is possible that the initial increase in open eye potentiation that we observed in adult EE-mice shortly after MD was driven by a similar reduction in firing rates of the inhibitory cells resulting in turn in a higher firing rate of excitatory neurons. However, a longer period of MD (7 days) might homeostatically restore the inhibition levels to normal, leading to an overall reduction in evoked firing rates.

Although EE-raised mice displayed OD-plasticity into adulthood [8], it was unknown whether this positive effect of EE on OD-plasticity would be maintained if mice are transferred to SCs. In fact, we found that OD-plasticity was rapidly lost under these circumstances. Thus, continued “enrichment” was needed to promote OD-plasticity in adult EE-mice and a surprisingly short exposure to a more deprived environment was sufficient to reverse the plasticity promoting effect of EE.

Among the factors previously described to be affected by EE compared to SC-raising is the neurotransmitter serotonin [7]. In particular, serotonin levels were elevated in EE-rats and administration of the selective serotonin reuptake inhibitor fluoxetine mimicked the effects of EE in SC-raised rats [24]. However, fluoxetine did not preserve OD-plasticity in our EE→SC-mice, while adding a running wheel (RW) to the SCs prevented the loss of OD-plasticity. The lack of a plasticity promoting effect of the fluoxetine treatment is somehow at odds with the observations of Maya-Vetencourt, Sale [24]. How can this discrepancy be reconciled? One possibility is that we used mice and not rats; another possibility is that the achieved fluoxetine concentration in the cortex was not high enough or the treatment was not long enough to promote plasticity. In fact, we did not test whether the drug was present in the visual cortex, and also our dosage was lower than the 0.2mg/ml used in the Maya-Vetencourt, Sale [24] study. We decided to use the lower dosage because it was previously used in mice and had been shown to improve depression-like phenotype, increase BDNF levels and reduce corticosterone levels in other mouse experiments [25].

The success in preserving plasticity with a RW supports a previous study [28] in which juvenile-like OD-plasticity was observed both after raising mice in SCs with a RW and even after just 1 week of RW-experience concomitant with the MD [28]. Interestingly, a study comparing the effects of running and fluoxetine treatment in the hippocampus revealed that the survival of newly generated cells was enhanced by 200% after running but not fluoxetine treatment, while both running and fluoxetine increased the percentage of newborn cells that became neurons [39]. Furthermore, fluoxetine treated mice were reported to be less active in locomotion [39]. These observations support our current findings that physical exercise was able to preserve OD-plasticity in V1 whereas fluoxetine treatment was not.

In contrast to OD-plasticity, experience-enabled optomotor enhancements after MD were maintained in all EE→SC-mice. A similar dissociation between the two visual plasticity paradigms was already reported previously: ibuprofen treatment rescued abolished optomotor enhancements after stroke but not OD-plasticity [40]. Likewise, OD-plasticity was preserved after stroke in PSD-95 KO mice while optomotor enhancements were absent [41], underscoring that experience-dependent network changes underlying these two forms of visual plasticity are mediated by distinct neuronal circuits as suggested before [33, 40].

Summarizing, our data stress the importance of the housing conditions on experimental animals and its significant effects on brain plasticity. In particular, experience-dependent changes in V1-activation happened much faster in EE-mice compared to animals raised under the deprived conditions of a SC. Surprisingly, even a few days without the possibility of voluntary physical exercise in adult EE-mice (e.g. by transferring them to a SC) completely abolished OD-plasticity in V1 of these mice, while adding a running wheel preserved plasticity. Thus, even a small change in the housing environment like the addition of a running wheel can have beneficial effects on brain plasticity. In turn, housing mice in a constrained environment (like a SC), with less stimulation and no opportunities for voluntary physical exercise clearly leads to an earlier decline of OD-plasticity and slower experience-dependent changes in cortical activation.
